# Evaluation of a silicon carbide P–N diode for thermal neutron detection in a radiotherapy LINAC

**DOI:** 10.1038/s41598-025-13052-w

**Published:** 2025-08-20

**Authors:** Martín Pérez, Felipe Zamorano, Celeste Fleta, Carles Muñoz-Montplet, Diego Jurado-Bruggeman, Marcio Jiménez, Pablo Guardia, Gina Grabulosa-Morera, Roger Morales-Pérez, Gemma Rius, Philippe Godignon, Giulio Pellegrini, Consuelo Guardiola

**Affiliations:** 1 Institute of Microelectronics of Barcelona (IMB-CNM-CSIC), 08193 Cerdanyola del Vallés (Bellaterra), Spain; 2 Medical Physics and Radiation Protection Department, Catalan Institute of Oncology (ICO), 17007 Girona, Spain; 3 Institute of Materials Science of Barcelona (ICMAB-CSIC), 08193 Cerdanyola del Vallés (Bellaterra), Spain

**Keywords:** Neutron detectors, SiC, Radiotherapy, Radiotherapy, Electrical and electronic engineering

## Abstract

Accurate neutron detection in mixed photon-neutron and pulsed radiation fields is technically challenging, impacting industrial and medical applications. This paper presents the first measurements of thermal neutrons in conventional radiotherapy accelerators using a silicon carbide (SiC) P–N diode with different neutron converters. SiC detectors enable real-time estimation of secondary thermal neutron contributions, crucial for emerging radiotherapy techniques requiring precise neutron fluence monitoring. Beyond medical applications, the presented detectors show potential for neutron dosimetry, radiation monitoring, nuclear safety, and scientific research. The SiC diode active detection layer is less than 30 µm thick, and provides excellent gamma rejection ($5\times 10^{-8}$), allowing discrimination of neutrons-induced events in mixed radiation fields. Experimental tests conducted on a TrueBeam radiotherapy LINAC demonstrated a thermal neutron detection efficiency of (4.32 ± 0.02)% for a (50 ± 10) µm thick $^6$LiF neutron converter. The detector, placed at 1.2 m from the accelerator isocenter, was used to measure neutron fluences at different monitor unit (MU) rates, ranging from 100 to 600 MU/min, with the LINAC operating at 15 MV. Under these conditions, the detector exhibited good linearity, without saturation or dead time effects.

## Introduction

Radiotherapy (RT) has become one of the key therapies against cancer that uses ionizing radiation to kill tumor cells^1,2^. Conventional linear accelerators (LINACs), commonly used in this type of treatment, generate beams of high-energy particles, including electrons and photons. When operating in photon mode, the primary electron beam strikes the LINAC target, typically made of gold or tungsten. If the accelerator is used to generate photons with energies greater than 8 MeV, secondary neutrons can also be produced in the LINAC head through photonuclear reactions. These reactions are associated with the giant dipole resonance in various LINAC structural components, such as the target, primary collimator, jaws, and multileaf collimator^3,4^. The production of secondary neutrons can be relevant when a large number of monitor units (MU) are used during treatment, for example in intensity-modulated radiotherapy (IMRT)^5^. The radiation produced outside the treatment field can result in peripheral doses that may affect healthy tissues surrounding the treated area^6–10^. Secondary neutrons can contribute to an unwanted dose of radiation to healthy tissues, generating an additional risk for the patient and medical staff. Thus, accurate detection and characterization of these neutron fields are essential to evaluate and mitigate their impacts.

Assessing the secondary dose resulting from neutron contamination requires knowledge of the neutron fluence within the treatment room. To estimate the biological impact, the absorbed dose must be adjusted using the radiation weighting factor (W$_R$), which accounts for the likelihood of stochastic radiation effects. The value of W$_R$ depends on the type of particle and its energy. For neutrons, the W$_R$ varies with energy, ranging from approximately 2.5 for slow neutrons to about 20 for fast neutrons. This is significantly higher than the W$_R$ of 1 assigned to both photons and electrons, as established by the International Commission on Radiological Protection (ICRP) in its 1992 and 2007 recommendations^11,12^. Likewise, as neutrons pass through the patient’s body, which is largely composed of light elements, they interact with the tissue nuclei through scattering and absorption. These interactions can lead to processes such as moderation, recoil, or neutron capture, all of which have the potential to leave an ionized track and cause tissue damage. Despite the inclusion of patient radiation protection in the treatment optimization guidelines by ICRP^12^, neutron levels remain significantly high. Therefore, it is important to assess the neutron radiation field within the treatment rooms to minimize the risk of secondary tumors.

Active characterization and/or monitoring of radiotherapy environments is challenging because of the pulsed nature of the mixed photon-neutron fields and the intense photon background. As a result, the use of passive neutron detectors has been recommended to avoid instrumental problems that arise with active devices, such as signal pile-up (AAPM 1986)^13^. Previous passive methods have relied on techniques such as foil activation within Bonner spheres^14–17^, TLD dosimeters^18–20^, and superheated bubble emulsions^21^. Although these methods are effective, they are time-consuming, as the detectors need to be analyzed in external laboratories after exposure, limiting their use in clinical settings. In contrast, active detectors can provide real-time information but face two primary challenges: (i) the photon fluence inside the treatment room can be extremely high, making it difficult for standard detectors to distinguish between neutron signals and the high photon background, and (ii) the mixed photon-neutron fields are pulsed, with frequencies in the hundreds of hertz, which can lead to signal pile-up from the high photon radiation. These factors have prevented the widespread use of real-time or active neutron field monitoring in RT rooms.

There are three main types of active detector that operate on the basis of the ionization produced by charged particles in matter: gas, scintillator, and semiconductor detectors. The most common gas detectors, proportional $^3$He tubes, are very effective in detecting thermal neutrons due to their high sensitivity, although their availability is limited and expensive^22^. Scintillator detectors are also used, generating light when interacting with neutrons, but they have low-energy resolution and usually have difficulties discriminating neutron-induced events from those produced by other types of particles. In addition, semiconductor detectors are widely used due to their compactness, which originates from the high density of the semiconductor material. This allows a short range of the nuclear reaction products, but also increases the probability of interaction with gamma rays. Moreover, conventional silicon detectors can be affected by structural damage, as a result of prolonged exposure to intense radiation, limiting their durability in high-energy applications.

Silicon carbide (SiC) has emerged as a promising material for neutron detection in radiological environments due to its remarkable properties, such as thermal stability and durability under extreme conditions^23^. It is also considered a promising semiconductor material for radiation-hardened dosimeters^24–26^. One of the key features of SiC is its wide bandgap of 3.27 eV, which reduces the leakage current and makes it insensitive to visible light. SiC also outperforms silicon in terms of radiation resistance, as it has a higher displacement energy threshold. On top of that, SiC exhibits better tissue equivalence. A particularly noteworthy property is its lower signal yield per deposited dose (425 pC$\cdot$mGy$^{-1}\cdot$mm$^{-3}$ for 4H-SiC), compared to silicon, which has a yield of 644 pC$\cdot$mGy$^{-1}\cdot$mm$^{-3}$. This characteristic makes SiC an ideal candidate for dosimetry in environments with ultra-high dose pulsed radiation or for direct beam monitoring, where traditional silicon diodes might saturate due to large instantaneous dose deposits in the semiconductor. Silicon carbide detectors can not only operate in high-radiation environments, but also exhibit higher efficiency in neutron detection compared to other traditional materials.

In recent years, the Institute of Microelectronics of Barcelona (IMB-CNM-CSIC) has developed its own technology for SiC radiation detectors. IMB-CNM-CSIC produced four-quadrant SiC photodiodes for beam position monitoring, which showed very good performance under varying temperature conditions and in the presence of visible light. These photodiodes also demonstrated superior radiation hardness for particle detection compared to traditional silicon detectors^23,27^. Additionally, 4H-SiC P-N junction diodes (PNDs), specifically designed for plasma diagnostic systems to detect alpha particles in future D-T fusion reactors, exhibited no degradation in their spectrometric performance when irradiated with 3.5 MeV alpha particles at room temperature, even at fluences up to $10^{11}$ cm$^{-2}$. These diodes maintained excellent energy resolution ($\le$2%) at temperatures up to 450$^\circ$C^28^. Recently, IMB-CNM-CSIC developed and manufactured a series of SiC-based diodes specifically designed for ultra-high dose rate (UHDR) dosimetry, with typical dose rates exceeding 40 Gy$\cdot$s$^{-1}$, as part of the EMPIR-UDH Pulse project^29^. These diodes successfully met the requirements for relative dosimetry in UHDR pulsed electron beams, handling doses up to 11 Gy per pulse and pulse durations ranging from 0.5 to 3 µs at 20 MeV. Furthermore, they demonstrated excellent radiation resistance, with only a minimal long-term decrease in sensitivity (0.018% per kGy) after exposure to accumulated electron doses^24^.

In this work, we evaluated the response of these novel SiC diodes under realistic radiotherapy room conditions for the first time. We also simulated the device response using TCAD Sentaurus^30^ and PHITS^31^. Additionally, we assessed the diode performance with four different thermal neutron converters and examined its response at different dose rates.

## Methods

### Silicon carbide detector

**Fig. 1:**
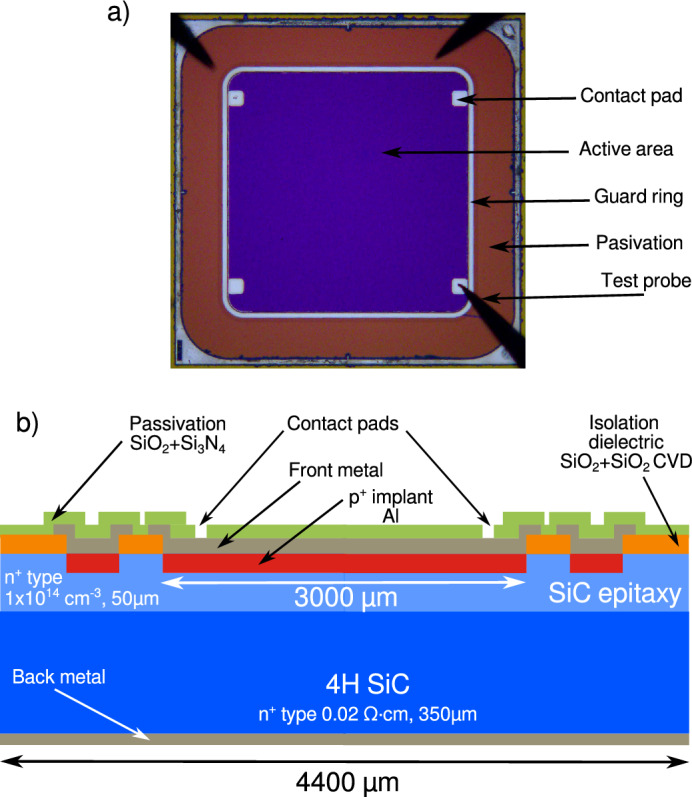
(**a**) Image of the detector obtained with an optical microscope. The detector was connected to the printed circuit board using a single bonding wire. (**b**) Scheme of the detector cross-section at the contacts pad position. Lateral dimensions and thicknesses not to scale.

Silicon carbide diodes were designed and fabricated at the IMB-CNM-CSIC on 350 µm thick 4H-SiC wafers with N-doped epitaxial layer of 50 µm with a nominal doping concentration of 1$\times$10$^{14}$ cm$^{-3}$. Figure 1a shows an image of the detector employed in this work. The picture was obtained with an optical microscope during an electrical characterization at a probe station and includes indications for the different elements of the device (e.g., contact pads, active area, guard ring, and passivation layer). Figure 1b depicts the corresponding scheme of the device cross-section. The SiC diode belongs to the same fabrication batch used in previous neutron measurements (Pérez et al.^32^). It features an active area of 3$\times$3 mm$^2$. In order to create the P-N junction, a highly doped P$^+$ layer was formed by Al ion implantation. A SiO$_2$/Si$_3$N$_4$ passivation layer was deposited on the surface of the diode to protect it from environmental damage. The front metal contact consists of a multilayer structure made of Ti/Al/Ti/Ni, and the back metal contact is composed of a multilayer structure of Ti/Ni/Au.

### Readout electronics

**Fig. 2:**
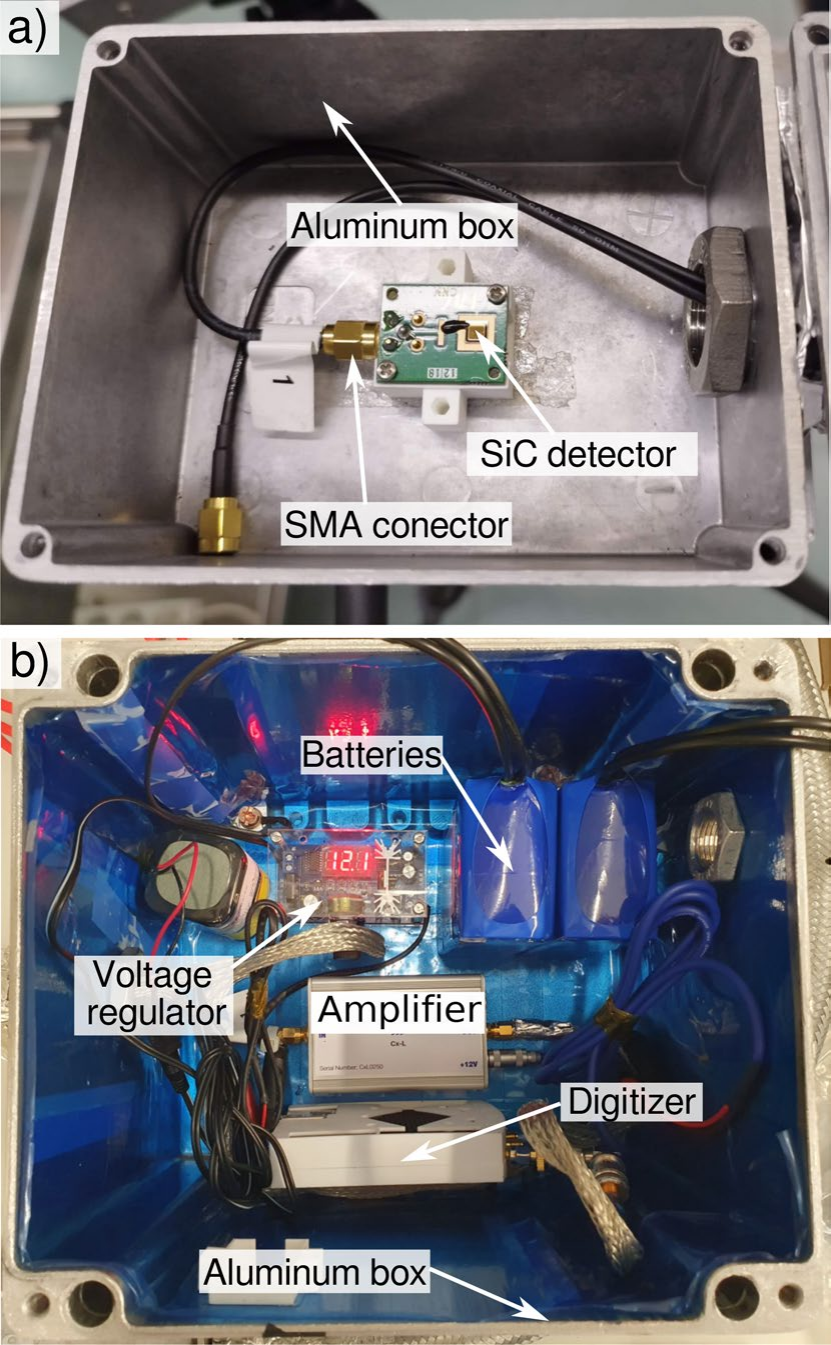
(**a**) Silicon carbide diode mounted on a customized PCB with a SMA connector inside an aluminum box. (**b**) Readout electronics consisting of a Cividec CxL0250 charge amplifier and a RedPitaya STEMlab 125-14 board. The charge amplifier was powered by batteries and a voltage regulator to eliminate power line pick-up noise.

The SiC diode under test was mounted on a customized printed circuit board (PCB), see Fig. 2a. The diode was placed inside a 2 mm thick aluminum box, which served as an electromagnetic shield. The thermal neutron transmission through the box walls is more than 97%^33^. The detector was connected to the readout electronics via a shielded cable with SMA connectors. A plastic base was used to electrically isolate the PCB. The readout electronics were housed in a separate metal box (see Fig. 2b) to keep them away from the irradiation zone. The pulses generated by the detector were amplified using a Cividec CxL0250 composed of a charge amplifier and a pulse shaper^34^. The amplifier output has a Gaussian shape, with a full width at half maximum (FWHM) of 700 ns, a rise time of 249 ns, and a gain of 12.6 mV/fC. The pulses were digitized using a RedPitaya STEMlab 125-14 board, which features a sampling rate of 125 Ms/s and a 14-bit ADC resolution^35^. The amplifier was powered by batteries and a voltage regulator to prevent pick-up noise from the power line. To enhance gamma rejection, the detector was biased with 0 V, at this voltage the measured capacity was 175 pF. The thickness of the depletion region (*W*) as a function of capacitance (*C*) is given by the following equation^36^:

1 $$\begin{aligned} W=\frac{\epsilon A}{C}. \end{aligned}$$

where *A* is the diode area (0.09 cm$^2$), and $\epsilon$ is the permittivity of SiC (8.588$\times 10^{-11}$ F/m) resulting in a depletion thickness of 4.42 µm.

### Neutron converters

Neutrons do not have electric charge, and therefore, do not interact with the orbital electrons of a medium via the Coulomb force. As a result, they do not directly produce primary ionization. Instead, neutrons interact with matter through nuclear forces. These interactions occur primarily through scattering and capture (or absorption) by atomic nuclei. When a neutron is captured, it triggers the emission of radiation from the target nucleus or the release of heavy ions and/or fundamental particles. One common method for detecting thermal neutrons involves the use of neutron converters (NCs). These converters are made of materials with a high neutron absorption cross-section, which generate charged particles upon interaction with thermal neutrons. Two commonly used materials for this purpose in solid-state detectors are $^6$Li and $^{10}$B. Regarding the boron element, the isotope with the highest cross section is $^{10}\text {B}$, which interacts with neutrons through the reaction^37^:

2 $$\begin{aligned} \text {n}+^{10}\text {B}\rightarrow {\left\{ \begin{array}{ll} ^{7}\text {Li} \ (1.015 \ \text {MeV}) + \alpha \ (1.777 \ \text {MeV}),~\text {6}\%,\\ ^{7}\text {Li} \ (0.840 \ \text {MeV}) + \alpha \ (1.470 \ \text {MeV})+\gamma \ (0.48 \ \text {MeV}),~\text {94}\%. \\ \end{array}\right. } \end{aligned}$$

The first reaction leads to the ground state, while the second leads to the excited state. The branching fractions for these reactions are 6% and 94%, respectively. The reaction has a Q-value of 2.792 MeV. The $^{10}\text {B}$ isotope has a thermal neutron capture cross-section of 3840 barns.

Considering lithium, the isotope with the highest neutron cross-section is $^6$Li, which reacts with neutrons according to the reaction:

3 $$\begin{aligned} \text {n}+ ^{6}\text {Li} \rightarrow \, ^{3}\text {H}\ (2.73\ \text {MeV}) + \alpha \ (2.05\ \text {MeV}), \end{aligned}$$

it emits a 2.05 MeV alpha and a 2.73 MeV tritium nucleus with a Q value of 4.78 MeV. By kinematic principles, these reaction products are oppositely directed when the energy of the incident neutron is low enough. The cross-section is 940 barns for thermal neutrons.

The SiC diode was characterized in conjunction with four different NC layers:

1. Two $^6$**LiF NCs** synthesized at IMB-CNM-CSIC using enriched Sigma-Aldrich 95% $^6$Li, following the fabrication process previusly detailed in Pérez *et al.*^32^. These converters layers were prepared by stirring a mixture at 300 rpm at 40$^\circ$C for 10 minutes, followed by a drying step at 30$^\circ$C for 2 hours in the presence of 10% PVDF. This process resulted in a viscous mixture with enhanced adhesion when applied to a planar surface, such as a plastic mica plate or a silicon substrate. A density of (0.87 ± 0.03) g/cm$^3$ was determined using a pycnometer, which aligns with the 0.78 g/cm$^3$ reported in^38^. The thicknesses of these NCs were measured using a KLA-Tencor P-15 contact profilometer^39^. According to McGregor *et al.*^40^ and Tchouaso M.T.^41^, the optimal thickness for a pure $^6$LiF NC ranges between 17 and 27 µm. Based on this, we employed the NC with a thickness of (20 ± 5) µm. However, since the fabricated layers are not perfectly uniform and the $^6$Li content is 95%, we also used a thicker layer of (50 ± 10) µm.
2. An **LTO layer** specifically synthesized for this application at the Institute of Materials Science of Barcelona (ICMAB-CSIC). This layer represents the first attempt at developing a method for depositing lithium-based conversion layers with uniform thickness over the surface of SiC devices during the fabrication process in the IMB-CNM-CSIC clean room. This NC has a thickness of (7 ± 1) µm, and was made of a mixture of LiTiO$_2$ and Li$_4$Ti$_5$O$_{12}$. The preparation of lithium titanate nanoparticles was adapted from the work of Li *et al.*^42^ and Tang *et al.*^43^. Briefly, a first solution consisting of 3 mL of titanium (IV) butoxide, 3 mL of ethanol, and 9 mL of methanol was homogeneously stirred in a beaker. A second solution containing 21 mL of acetonitrile, 9 mL of ethanol, and 0.25 mL of ammonia was magnetically stirred for 30 minutes. The first solution was added dropwise into the second one under continuous stirring and kept under the same conditions for an additional 2 hours at room temperature. The resulting product was separated by centrifugation and cleaned with ethanol. Next, 0.3 g of the obtained product was mixed with 0.3 g of LiOH$\cdot$H$_2$O in a 25 mL ethanol-water solution (1:1 ratio) and transferred into a 25 mL Teflon-lined stainless-steel autoclave, which was then placed in an oven at 180$^\circ$C for 12 hours. The final powder was precipitated, washed with water by centrifugation, and dried in an oven at 60$^\circ$C for 24 hours before being dispersed in ethanol and drop casted onto the substrate.
3. A **BE10 screen** manufactured by Dosirad^44^, consisting of a mixture of 93% $^{10}$B-enriched B$_4$C glued on a 100 µm thick polyester base. The thickness of this conversion layer was measured using a KLA-Tencor P-15 contact profilometer^39^ obtaining (45 ± 5) µm.

### Electric simulations

The SYNOPSYS TCAD Sentaurus^30,45,46^ software was used to simulate the electrical characteristics of the SiC diode. This software enables the modeling of semiconductor device behavior, the simulation of manufacturing processes, and the design of device structures. In addition, TCAD can be used to analyze the electrical characteristics of semiconductors in response to external boundary conditions of electrical, thermal, or optical nature.

The silicon carbide diode (Fig. 1) was simulated using three distinct regions: Firstly, the substrate, which has low resistivity and is doped with a nitrogen concentration of $1\times 10^{18}\, \text {cm}^{-3}$, where the backside electrode (N^+^) is placed. Secondly, the N-type epitaxial layer, which has a thickness of 50 µm and a nitrogen concentration of $1\times 10^{14}\,\text {cm}^{-3}$. Finally the top electrode (P^+^), which was simulated employing a Gaussian profile of aluminum implantation, with a peak concentration of $7.7 \times 10^{19} \, \text {cm}^{-3}$. Additionally, a dielectric isolation layer of 1 µm-thick SiO_2_ was included. To estimate the charge collected by the detector at 0 V at different depths, the HevyIon model was used to introduce charge clouds of $2 \times 10^{-3} \, \text {pC}/\mu \text {m}$ in short segments (0.5 µm) with a lateral Gaussian distribution of 0.5 µm. Charge collection was evaluated using an integration time of 2 µs. Since the depleted region was approximately 4.4 µm, the selected regions were both inside and outside of this area. The charge collection data obtained from these simulations were subsequently used to model the deposited energy in PHITS.

### PHITS Monte Carlo code

The response of the detector was simulated using the PHITS code based on the Monte Carlo (MC) method. This tool was developed in collaboration with the Japan Atomic Energy Agency (JAEA) and several international research institutes^31^. For this study, simulations were performed using PHITS version 3.34, with the updated JENDL-5.0 nuclear data library.

To simulate the neutron fluence into the LINAC bunker, the code parameters were fine-tuned to replicate the geometry of the Varian TrueBeam head operating at 15 MV^50^. Based on the blueprints of a Varian Clinac 2100, provided in the MC Data package distributed by Varian to its users, modifications were introduced in line with previous research (M. Sumini *et al.*^47^): The flattening filter was replaced from tungsten to a copper-based material. Additionally, head shielding was incorporated into the model presented by Quoc *et al.*^48^. The simulation of photo-neutron production and particle transport through the bunker was carried out in a two-step process. In order to speed up the computing time, a lower energy cut-off was set at 6 MeV for electrons, positrons and photons and the complete photo-neutron spectrum was tallied on the inner surface of a spherical shell surrounding the LINAC head, generating a Phase-Space File (NDS-IAEA^49^). In the second step, this file was used as the source for the particle diffusion into the treatment room. The room geometry was designed to match the bunker layout, with concrete walls made of Ledite XN-240 (3.8 g$\cdot$cm$^{-3}$) and Ledite XN-288 (4.8 g$\cdot$cm$^{-3}$) specialized heavy concrete materials supplied by Atomic International (USA).

The detector was placed at the same location as in the experimental setup, incorporating aluminum housing, PCB, plastic holder, diode specifications, and conversion layers. Particle energy deposition was calculated via the [T-Deposit] tally, which provides the energy deposition distribution.

### Energy calibration and gamma rejection

**Fig. 3:**
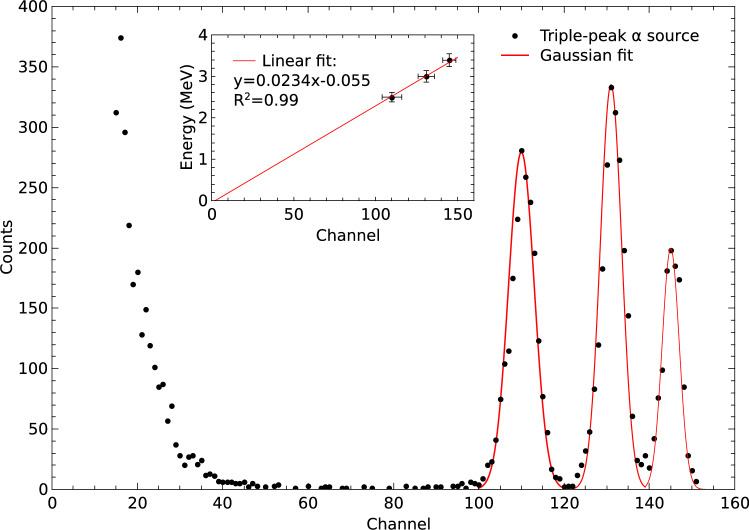
Spectrum obtained in an irradiation with a triple-peak alpha source. The red curve depicts the Gaussian fits of the measured peaks. The inset shows the energy calibration curve of the detector.

In order to perform an energy calibration, the SiC diode was irradiated with a triple-peak alpha source of $^{239}\text {Pu}$, $^{241}\text {Am}$, and $^{244}\text {Cm}$ with main emissions of 5.15, 5.48, and 5.9 MeV respectively. The source was positioned at a distance of 22 mm from the sensor. Under these conditions, the alpha particles lose energy in the air and the insulating layers of the device, resulting in maximum deposited energies of (2.5$\pm 0.1$), (3$\pm 0.1$), and (3.39$\pm 0.15$) MeV respectively. The spectrum obtained in the calibration with the triple-peak alpha source is presented in Fig. 3. It was possible to resolve the three peaks produced by the $\alpha$ particles. The red curve represents the Gaussian fit of the peaks, while the inset in the figure shows the sensor calibration curve.

To determine the gamma rejection of the detector, we performed an irradiation using photons of 662 keV emitted by a $^{137}$Cs source with an activity of 67.1 mCi. The bare detector (without NC) was placed at 40 cm from the source and irradiated for 30 minutes. In these measurements, a lower-level discriminator (LLD) of 50 keV was used. During irradiation, no events were detected, leading to the conclusion that, under these experimental conditions, the gamma rejection factor is greater than 5$\times$10$^{-8}$.

### Experimental setup

**Fig. 4:**
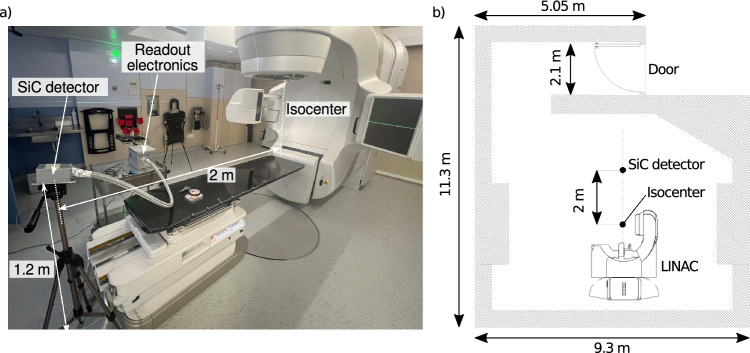
(**a**) Picture of the experimental setup in the TrueBeam LINAC room at ICO-Girona. (**b**) Plan of the bunker that shows the position of the SiC detector with respect to the accelerator isocenter.

In order to evaluate the performance of the detector, we carried out a series of irradiations employing a 15 MV flattened-filter (FF) beam from a Varian TrueBeam radiotherapy accelerator, which is the current platform of multi-energetic Varian LINACs. The experiments were done at the Radiation Oncology Department of the Catalan Institute of Oncology (ICO)-Girona. We employed a static 10$\times$10 cm$^2$ field with a gantry position of 0º. No phantom was used during the experiment. A series of irradiation runs were carried out to test the detector linearity and its response as a function of the dose and dose rate. The total dose ranged from 100 to 1000 monitor units (MU) and the dose rate varies between 100 and 600 MU per minute. Figure 4a shows a picture of the experimental setup, the detector was placed at a distance of 2 m from the accelerator isocenter at a height of 1.2 m. Figure 4b depicts a plan of the accelerator bunker indicating the position of the detector.

## Results and discussion

**Fig. 5:**
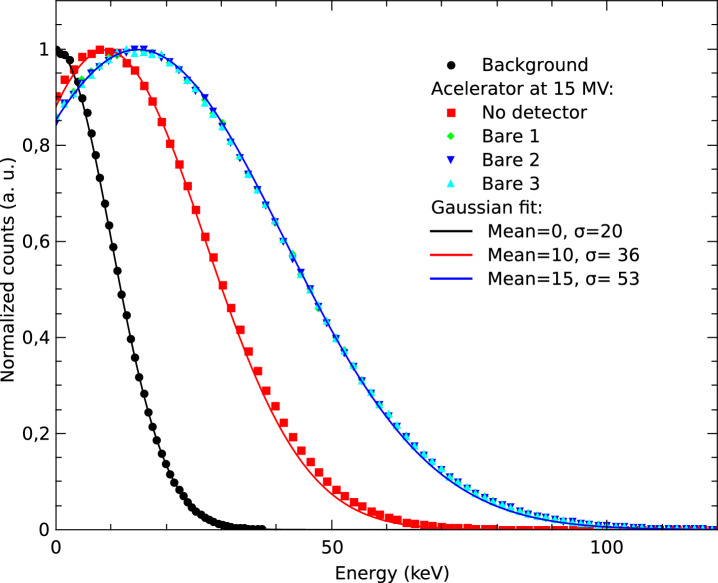
Background and detector noise measurements (dotted curves). Gaussian fits (continuous lines).

This section presents the response of the SiC neutron detector. Figure 5 presents a measurement of background and detector noise (dotted curves) carried out in the accelerator bunker. Firstly, we performed a background measure (black dots) with the bare detector and the LINAC off. The noise presents a mean value of 0 keV and a standard deviation ($\sigma$) of 20 keV. Secondly, we verified the noise level produced by the electromagnetic interference generated by the accelerator with an irradiation of 100 MU at 15 MV without connecting the detector to the amplifier (red dots). It is possible to observe that the mean value of the noise is 10 keV, with a $\sigma$ of 36 keV. Third, we performed 3 irradiations of 100 MU at 15 MV with the bare detector (without NC), in these conditions, the measured signal is due to detector noise and background, as well as electromagnetic interference within the bunker, and signal produced by photons emitted by the accelerator. The continuous lines of Fig. 5 show Gaussian fits of the experimental data. The spectra obtained in the three measurements are equivalent, showing a mean value of 15 keV and a $\sigma$ of 53 keV. Based on the results of these measurements, a lower-level discrimination (LLD) threshold of 150 keV was established for neutron detection.

The SiC diode, covered with a (50 ± 10) µm-thick $^6$LiF NC layer, was tested under an irradiation with 300 MU of 6 MV photons at a rate of 600 MU/min. Under these conditions, no neutrons are produced and, as expected, the detector did not register any events.

**Fig. 6:**
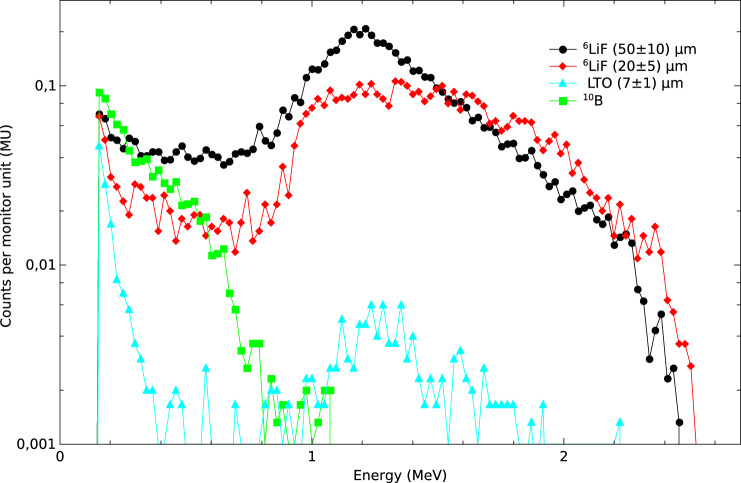
Comparison of the spectra obtained with the different conversion layers. The measurements were performed with the accelerator operating at 15 MV.

Figure 6 shows the spectra obtained with all NCs performed with the accelerator operating at 15 MV. In the Li-based NCs, the registered events are produced by tritium ions of 2.73 MeV and alpha particles of 2.05 MeV generated by the $^6$Li(n,$\alpha$)$^3$H reaction. In this case, it can be observed that the spectra corresponding to the $^6$LiF NCs exhibit a peak at approximately 1.2 MeV. This is because most of the 2.7 MeV tritium ions generated after neutron interaction lose energy in the neutron converter and the insulating layers of the chip before reaching the detector active volume. In the case of $^6$LiF NCs, the maximum energy deposited by the tritium ions is approximately 2.5 MeV. Most of the detected events in the $^{10}$B NC are produced by alpha particles of 1.47 MeV product of the $^{10}$B(n,$\alpha$)$^7$Li reaction. In this case, the maximum energy deposited by the alpha particles is around 1 MeV, and it is not possible to distinguish any peak.

It is possible to observe that the (50 ± 10) µm thick $^6$LiF CL produces the highest counts per MU, followed by the (20 ± 5) µm thick $^6$LiF CL. In the case of the LTO NC, the number of detected events is an order of magnitude lower. On the one hand, this is due to the use of natural lithium, which contains only 7.59% of $^6$Li. On the other hand, the low counting rate is also attributed to the NC thickness being lower than the optimal range, that for $^6$LiF is between 17 and 27 µm. The presence of lithium in the LTO NC can be identified by the peak at approximately 1.2 MeV, which coincides with the peak positions observed in the $^6$LiF NC. Additionally, a significant number of counts is observed at lower energies (150–400 keV), possibly due to tritium and alpha particles losing energy in the titanium present in this NC. Since we obtained the highest detection efficiency with the $^6$LiF NC, we performed the following measurements and simulations only considering these layers.

**Fig. 7:**
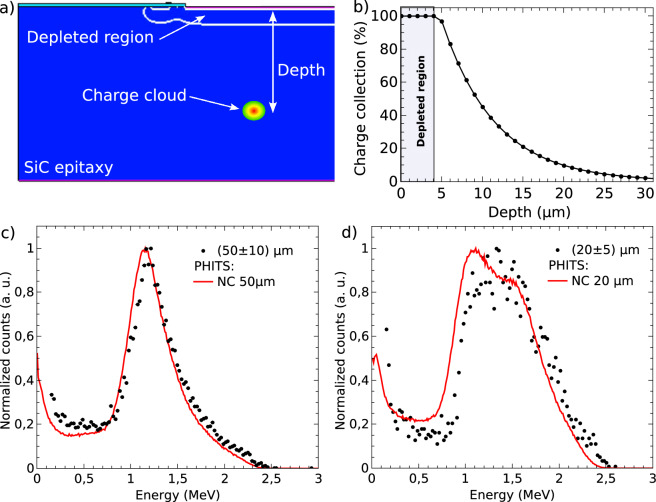
(**a**) Section of the SiC diode geometry employed to perform the TCAD simulation. (**b**) Charge collection efficiency (CCE) as a function of the depth. (**c**) and (**d**) results of the simulations (lines) compared with the experimental data (dots) for the $^6$LiF NCs of (50 ± 10) µm and (20 ± 5) µm respectively.

Taking into account the electrical measurements performed after the device fabrication, for a bias voltage of 0 V, the junction capacitance is 1.75$\times$10$^{-10}$ F. Therefore, the thickness of the depletion region for this bias voltage is 4.41 µm. However, under these conditions, the effective thickness of the detector active volume is greater than that of the depletion region. This occurs because a fraction of the electron-hole pairs generated in the detector epitaxial region diffuse toward the depletion region and are collected by the electric field present in this area, thereby producing pulses with greater amplitude. This behavior was accurately modeled employing TCAD electrical simulations, with the parameters detailed in the Methods section. To analyze the charge collection efficiency (CCE) as a function of distance from the depleted region, a charge cloud of 0.5$\times$0.5 µm and 100 kV/µm was defined at various positions. The CCE values as a function of depth were determined by comparing the collected charge with the maximum value obtained at the position closest to the p$^+$ electrode within the depleted region. Figure 7a shows a section of the geometry used in the TCAD simulations, where the limit of the depletion region and one of the charge cloud positions used to simulate the CCE can be observed. Figure 7b presents the CCE as a function of depth: on the one hand, it is possible to observe that within the depletion region (from 0 to 4 µm) the CCE is 100 %. On the other hand, the CCE presents an exponential behavior behind the depletion zone, it is possible to see that there is a non-negligible charge collection between 4 to 30 µm.

The CCE results were incorporated into the PHITS simulations. To calculate the absorbed energy, the [T-Deposit] tally was used, implementing a weighted summation approach to derive the energy spectra. In this method, the energy deposited in the *i*-th region for each particle history, denoted as $E(\text {history}, i)$, is multiplied by the corresponding charge collection efficiency at that depth, $\text {CCE}(i)$. The resulting values are then summed to obtain the total absorbed energy. The 50 µm-thick SiC detector was modeled as an array of regions (voxels) with a depth resolution of 1 µm. Figures 7c and d present the results of the simulations (curves represented by lines) compared to the experimental data (dots) for the $^6$LiF NCs of (50 ± 10) µm and (20 ± 5) µm respectively. PHITS simulations were performed employing conversion layers with uniform thickness. The results exhibit good consistency with the experimental data for both NCs. Table 1 presents a comparison between the FWHM and the mean values of the peaks shown in Fig. 7a and b. It also reports the percentage differences between the measured and simulated values. The maximum percentage difference observed is 11.3%, which corresponds to the FWHM of the 20 µm NC layer. The observed discrepancies may stem from non-uniformities in NC thickness and minor inconsistencies between the measured and simulated charge collection efficiency.

**Table 1:** FWHM and mean values from experimental data and simulations for the peaks in Fig. 7c and d, along with their relative percentage differences.

NC thickness	Parameter		Experimental (MeV)	Simulated (MeV)	Difference (%)
50 ${\upmu }$m	FHWM		0.51 ± 0.05	0.50 ± 0.05	1.3
	Mean value		1.18 ± 0.02	1.15 ± 0.05	2.5
20 ${\upmu }$m	FHWM		0.86 ± 0.07	0.97 ± 0.01	11.3
	Mean value		1.4 ± 0.9	1.3 ± 0.8	7

**Fig. 8:**
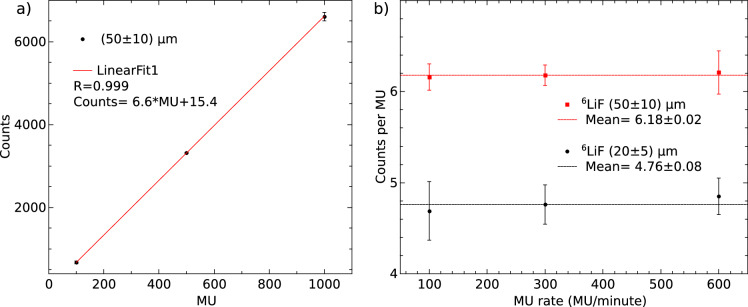
(**a**) Number of registered counts as a function of the total MU emitted by the LINAC measured with the (50 ± 10) µm thick $^6$LiF NC. (**b**) Counts per MU as a function of the MU rate for the $^6$LiF NCs.

Figure 8a presents a plot of the number of registered counts as a function of the total MU emitted by the LINAC for the $^6$LiF NC with a thickness of (50 ± 10) µm. The fit shown by the red curve indicates that the detector response remains linear within the range of 100 to 1000 MU. Figure 8b displays a plot of the counts per MU as a function of the MU rate for the $^6$LiF NCs. The data show that the counts per MU remain constant for both NCs between 100 and 600 MU per minute, with mean values of 6.18 ± 0.02 and 4.76 ± 0.08 for the (20 ± 10) µm and (50 ± 10) µm $^6$LiF CLs, respectively. This result indicates that, within this MU rate range, neutron counting is not affected by saturation or dead-time effects.

From the PHITS simulations of the LINAC bunker, we estimate that the total neutron fluence at the detector position is $1.7(0.3)\times 10^6\,\text {n}\cdot \text {cm}^{-2}\cdot \text {Gy}^{-1}$. Zamorano *et al.*^50^ present a simulation of the neutron flux at the exact position of the detector, which shows a peak in the thermal range at around 70 meV and another corresponding to fast neutrons at approximately 0.3 MeV. About 9.4% of the simulated flux ($1.6 \pm 0.3 \times 10^5$ n$\cdot$cm$^{-2}$ $\cdot$Gy$^{-1}$) corresponds to neutrons in the thermal energy range. Based on the counts per MU shown in Fig. 8b, the estimated detection efficiency is $(4.32 \pm 0.02)\%$ for the $(50 \pm 10)$ µm thick ^6^LiF NC and $(3.33 \pm 0.06)\%$ for the $(20 \pm 10)$ µm thick ^6^LiF NC. The thermal neutron cross-section of ^6^Li is approximately 1000 barns. In contrast, in the energy range of the fast neutrons present in the room, the cross-section is more than two orders of magnitude lower. Consequently, the total number of events produced by 0.3 MeV fast neutrons in the bunker is also more than two orders of magnitude lower than that produced by thermal neutrons.

## Conclusions

For the first time, thermal neutron fluences were successfully measured in a radiotherapy room using a neutron detector based on a silicon carbide P–N diode covered with different neutron converters. On the one hand, $^{10}$B is not the most suitable option as a NC for use in conjunction with this type of SiC diodes because the energy of the secondary particles emitted after its interaction with neutrons is low. Consequently, the energy deposited by these particles in the semiconductor active volume is close to the lower-level discriminator threshold, which, in this case, must be set higher than in other environments due to the high gamma-ray component and the electromagnetic noise. On the other hand, the energy of the products emitted after reactions between thermal neutrons and $^{6}$Li makes it the most suitable for pulse-height analysis and for discriminating neutron-induced events in mixed gamma+neutron fields.

From the neutron converter comparison presented in Fig. 6, it is possible to observe that the 50 µm thick $^6$LiF NC exhibits the highest efficiency, followed by the 20 µm thick $^6$LiF NC. Future work will focus on implementing the deposition of LTO neutron converters onto the surface of SiC diodes during the fabrication process. This deposition method will improve the uniformity of the neutron converter thickness and eliminate possible air gaps between the NC and the passivation layer of the sensors. Although LTO NC exhibits a lower counting rate than LiF NC, its efficiency could be improved by using enriched $^6$Li in the synthesis process.

Due to diffusion in the diode epitaxial layer, the effective thickness of the detector is greater than that of the depleted region, this behavior could be modeled using TCAD electrical simulations and the PHITS Monte Carlo code. Although the diffusion phenomenon increases the effective thickness of the detector (simulations show that charge collection occurs up to 30 µm deep in the epitaxial region) the device maintains a good photon rejection factor. The photon contribution did not interfere with the measurements, and neutron-induced events were effectively discriminated through pulse-height analysis.

The simulated neutron fluences are consistent with the measured values reported by Domingo *et al.*^17^ for a similar 15 MV Varian LINAC. Their reported values range from $163.0(6.8)\times 10^4\,\text {n}\cdot \text {cm}^{-2}\cdot \text {Gy}^{-1}$ to $515.7(1.5) \times 10^4\,\text {n}\cdot \text {cm}^{-2}\cdot \text {Gy}^{-1}$. In addition, the thermal detection efficiency obtained is consistent with the theoretical values presented by McGregor *et al.*^40^ and with the previous results reported by our group in Pérez * et al.*^32^.

The neutron detectors implemented in this work offer promising capabilities for a wide range of applications, including neutron dosimetry, radiation monitoring, nuclear safety, and scientific research. This development represents an important step toward the design and production of innovative SiC neutron detector arrays at the IMB-CNM-CSIC clean room. The robustness and high radiation tolerance of SiC make these detectors suitable for measuring neutron fluences across different energy ranges, addressing key challenges in neutron detection.

Furthermore, SiC detectors are promising for FLASH radiotherapy because of their high radiation tolerance, fast response time, and low sensitivity to gamma radiation. These features make them well-suited for monitoring the extremely high dose rates delivered over very short periods in FLASH treatments^25^. Unlike conventional detectors, SiC devices can maintain performance under high cumulative doses, which is essential for accurate and reliable dosimetry in this emerging technique.

Additionally, this type of detectors can be employed for the measurement of out-of-field neutrons in radiotherapy beams using other types of ions such as protons, helium, carbon, and oxygen, to complement and/or replace other neutron detectors^51^. Owing to their robustness under high radiation environments, silicon carbide-based detectors also hold promise for neutron dosimetry in pulsed beams, where conventional detectors often suffer from saturation effects^52^.

Following these initial measurements with conventional beams, the focus will shift toward developing a detector matrix capable of measuring neutron fluences across different energy ranges in pulsed beams under FLASH conditions. Their ability to discriminate neutron signals from gamma backgrounds, combined with their compact and durable design, positions them as a promising solution for future advancements in medical physics and radiation protection.

## Data Availability

All data and the simulations of this work are available. There will not be restrictions to use the generated data after its publication, but external users will be asked to cite the source and/or the papers properly, and the redistribution of the data must be authorized by the corresponding author. The identity of the person accessing the data will be ascertained with a questionnaire, available at http://hdl.handle.net/10261/396531.
